# Aberrant Positivity for BCL6 Corepressor (BCOR) Immunohistochemistry in Undifferentiated Round Cell Sarcoma of the Kidney With Melanoma of the Ureter

**DOI:** 10.7759/cureus.87855

**Published:** 2025-07-13

**Authors:** Shuichi Ishibashi, Narimasa Funabashi, Daisuke Nino, Takeshi Taketani, Masaaki Hidaka

**Affiliations:** 1 Digestive and General Surgery, Shimane University Faculty of Medicine, Izumo, JPN; 2 Pathology, Shimane University Faculty of Medicine, Izumo, JPN; 3 Pediatrics, Shimane University Faculty of Medicine, Izumo, JPN

**Keywords:** ewing sarcoma, melanoma, sarcoma of the kidney, sarcoma with bcor genetic alterations, undifferentiated round cell sarcoma

## Abstract

BCL6 corepressor (BCOR)-rearrangement sarcoma, an undifferentiated round cell sarcoma, is rare, and its pathophysiology is diverse. A 14-year-old girl presented with a tumor in the left kidney with invasion of the left iliopsoas muscle, left renal vein, and inferior vena cava. An immunohistochemical analysis of the tumor biopsy specimen was positive for BCOR, but no BCOR gene aberrations were detected. Chemotherapy was administered according to the Ewing's sarcoma protocol. When the residual tumor was resected after chemotherapy, melanoma was found in the ureter, which was removed. At eight months after surgery, the patient relapsed and underwent another round of chemotherapy, followed by additional irradiation. These results suggest that some sarcomas without BCOR gene variants were found to be BCOR-positive by immunohistochemistry (IHC) and that melanoma and sarcoma coexisted.

## Introduction

BCL6 corepressor (BCOR)-rearrangement sarcoma was first identified by Pierron et al. in 2012 among 594 cases of undifferentiated round cell sarcoma (URCS) that morphologically resembled Ewing sarcoma (EWS), but lacked the canonical EWS RNA-binding protein 1-erythroblast transformation-specific family genes (EWSR1-ETS) translocation [1]. ESWR1-ETS fusion is a characteristic finding in EWS and has been referred to as Ewing-like sarcoma without ESWR1-ETS translocation [2]. In recent years, rapid genetic characterization of tumors has led to the recognition of some Ewing-like sarcomas as independent disease units; BCOR-related sarcoma is one of these. Briefly, URCS are a group of aggressive tumors composed of small, primitive-appearing cells. Many are genetically defined by EWSR1-ETS translocations, but some, like BCOR-rearranged sarcomas, lack this hallmark.

Melanoma is a malignant tumor composed of melanocytes, most commonly in the skin. Although it represents a small proportion of skin cancers, it accounts for the majority of skin cancer-related deaths owing to its aggressive nature and high potential for metastasis. Clinically, it often presents as a new or changing pigmented lesion, as assessed using the ABCDE criteria. The diagnosis is confirmed by biopsy, and treatment depends on stage, ranging from surgical excision to immunotherapy or targeted therapy. Recent advances, particularly in immunotherapy, have improved survival rates in advanced cases. Early detection is critical for achieving favorable outcomes.

In this report, we present the case of a girl diagnosed with URCS of the left kidney and melanoma of the ureter. BCOR immunohistochemistry was diffusely positive; however, genetic testing revealed no alterations in the BCOR gene. We aimed to highlight the clinical presentation, diagnostic approach, and therapeutic strategies and comprehensively review the current literature.

## Case presentation

**Figure 1:**
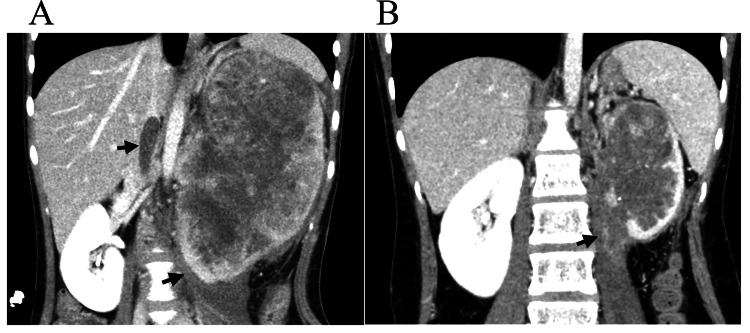
CT scan images. (A) Contrast-enhanced CT image obtained before chemotherapy demonstrating a large soft tissue mass (107 × 77 × 151 mm) originating from the left kidney, with invasion into the inferior vena cava (IVC) and left psoas major muscle. (B) Contrast-enhanced CT image obtained after chemotherapy showing a reduction in tumor size (59 × 56 × 101 mm), but psoas muscle invasion remained.

A 14-year-old girl with an unremarkable medical history presented to our hospital with a chief complaint of left upper abdominal pain that had persisted for three months. Blood tests showed elevated inflammatory markers, including a WBC count of 25,750/μg and a CRP level of 9.15 mg/dL, but no significant elevation of tumor markers (alpha-fetoprotein (AFP), human chorionic gonadotropin-beta (hCG-β), neuron-specific enolase (NSE), squamous cell carcinoma (SCC), carcinoembryonic antigen (CEA), and carbohydrate antigen 125 (CA125)). CT of the abdomen and pelvic CT revealed a 99 × 77 × 153 mm left renal mass with a tumor thrombus extending through the inferior vena cava (IVC) and psoas major muscle invasion (Figure 1A). 

**Figure 2:**
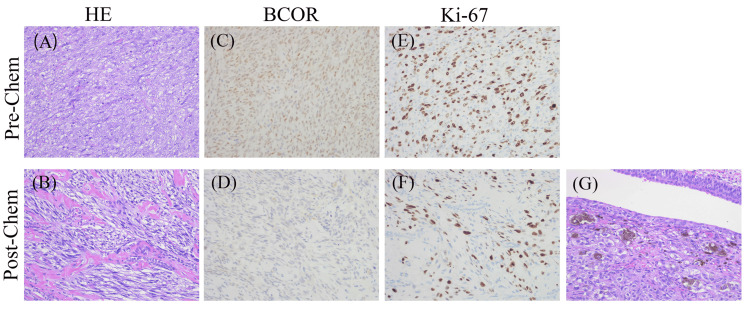
Histopathological and immunohistochemistry (IHC) images (×400). (A) Hematoxylin and eosin (HE) staining of the pre-chemotherapy biopsy revealed spindle cell infiltration into the vessel wall with a high nuclear-to-cytoplasmic ratio. (B) The post-chemotherapy specimens showed that much of the renal tissue had been replaced by fibrous tissue, and the therapeutic effect of chemotherapy was estimated to be >90%. (C)-(F) IHC staining demonstrated that BCOR and Ki-67 showed diffuse and strong pre-chemotherapy. However, BCOR became negative post-chemotherapy and the Ki index decreased from 40% to 3% after chemotherapy. (G) Hematoxylin and eosin staining revealed a melanoma component within the ureteral tissue of the resected tumor. This melanoma was positive for melanocytic markers S100, HMB-45, and Melan-A. Pre-chem: pre-chemotherapy, post-chem: post-chemotherapy, HE: hematoxylin-eosin stain, BCOR: BCL-6 corepressor.

Biopsy of the tumor showed diffuse spindle cells and aberrantly positive BCOR immunohistochemistry (IHC) (Figures 2A, 2C, 2E). We considered this tumor to be an Ewing-like sarcoma and chose chemotherapy according to the treatment strategy for EWS. The patient received four courses of neoadjuvant chemotherapy with a regimen of vincristine, doxorubicin, cyclophosphamide, ifosfamide, and etoposide (VDC-IE), and the tumor shrank (Figure 1B). Subsequently, the left kidney and part of the ureter were resected. The abdomen was opened through a left lateral abdominal incision, and the left renal tumor was removed. During surgery, a portion of the iliopsoas muscle was partially excised, and tumor invasion into the left renal vein was observed. The resected tumor showed decreased tumor cells on hematoxylin and eosin (HE) staining and IHC (Figures 2B, 2D, 2F), and surprisingly showed a melanoma component in the ureteral tissue (Figure 2G). Eight months after surgery, a local recurrence was detected during routine follow-up, and a tumor biopsy was performed, leading to a tissue diagnosis of recurrent sarcoma. She received six courses of chemotherapy (temozolomide, irinotecan) and radiotherapy (left side of the abdomen; dosage, 50 Gy/25 f). The patient was disease-free and well at six months after the treatment of recurrence. Sarcoma with BCOR genetic alterations was suspected. Next-generation sequencing (NGS) was used to search for genetic mutations in the BCOR gene comprehensively, but no genetic mutations were found (in addition, no EWSR1-ETS translocation was found).

## Discussion

URCS represents a new group of tumors defined by specific genetic abnormalities. According to the 2020 World Health Organization classification, URCS is a newly classified category of bone and soft tissue sarcoma [3]. This newly defined group includes EWS and small round cell sarcoma, previously known as Ewing-like sarcoma. Sarcomas with BCOR genetic alterations are classified as URCS. The histological features include variably round to spindled cells, with some cases dominated by spindle cell morphology. The individual cells contain uniform nuclei with fine chromatin and scant cytoplasm. IHC shows that the nuclear BCOR expression is highly sensitive, regardless of its fusion partner [3]. The BCOR gene, located at Xp11.4, encodes the ubiquitously expressed BCOR protein (interacts with BCL6) to enhance BCL-6-mediated transcriptional repression [4]. Physiologically, BCOR plays an important role in pluripotency by regulating cell differentiation and fate determination [1]. In bone and soft tissue sarcomas, BCOR gene mutations mainly occur through internal tandem duplications (ITDs), and BCOR-related fusions, such as BCOR-CCNB3, are found. In addition to CCNB3, BCOR has been reported to fuse with ZC3H7B, CIITA, MAML3, and KMT2D [5]. BCOR IHC is a highly sensitive marker for identifying round cell sarcomas with abnormal BCOR expression. Furthermore, other tumors such as clear cell sarcoma of the kidney (CCSK) share both histomorphology and genetic changes with these soft tissue tumors, as well as BCOR immunoreactivity [6]. BCOR-CCNB3 tumors occur primarily in the bone, whereas other BCOR-associated sarcomas arise in intra-abdominal soft tissues [6,7].

Motoi et al. [8] reported three cases of soft tissue sarcomas (temple mass, knee mass, and subcutaneous mass in the leg) without BCOR mutations, but with phenotypic and epigenetic characteristics consistent with BCOR-associated sarcomas. All three cases showed histological findings indistinguishable from those of BCOR-associated sarcomas, but were not accompanied by BCOR mutations. In our case, the tumor was positive for BCOR by IHC, and histological findings were indistinguishable from those of BCOR-associated sarcoma; however, a comprehensive analysis by NGS did not identify any BCOR gene or alterations. Aberrant positivity for BCOR IHC in URCS without BCOR gene mutations has never been reported in renal origin.

In one of these three cases, as in the present case, the tumor was resected after preoperative chemotherapy, recurrence was observed, and the patient was treated with postoperative chemotherapy and radiation therapy. These results suggest that there is a group of URCS patients who have BCOR-positive tumors on IHC but no BCOR gene aberrations on genetic testing.

There is no established treatment for patients with tumors positive for BCOR on IHC. Although there is no consensus on the optimal treatment due to a lack of clinical trials, the use of Ewing's sarcoma regimen (VDC-IE) is commonly reported, and chemotherapy is administered according to EWS [1,9,10]. The residual tumors were resected after preoperative chemotherapy, but recurrence was observed. Therefore, we administered postoperative chemotherapy with RT. Histological images showed a decrease in tumor cells and Ki-67, suggesting a favorable therapeutic response. In previous reports, a decrease in BCOR-positive cells was observed as a histological sign of favorable treatment response [11]. These results indicate that it is necessary to establish treatment for patients with the positive expression of BCOR on IHC and no detectable BCOR gene mutations.

In addition, a part of the ureteral tissue in this case contained a melanoma component. It has been reported to progress chronologically from malignant melanoma through undifferentiated sarcoma to rhabdomyosarcoma [12]. Patients in the group where rhabdomyosarcoma differentiation was detected in the nodules or distant metastases were significantly younger and predominantly female [10]. In the present case, melanoma was present in the ureter, which was not contiguous with the renal tumor. Although not a distant metastasis, the melanoma component may have developed in parallel with URCS or through a related dedifferentiation process. This suggests that there might be a group of URCSs arising from melanoma, although the mechanism of carcinogenesis is not clear.

## Conclusions

These observations suggest that certain URCSs lacking detectable BCOR gene alterations at the molecular level may nonetheless exhibit BCOR protein expression, as determined by IHC. Additionally, the findings imply the potential histopathological coexistence of melanoma and URCS components in anatomically distinct lesions, indicating a diagnostically challenging and possibly biologically complex tumor entity. In the future, we would like to further verify the molecular biological characteristics of the tumor.
